# Radiation exposure dose and influencing factors during endoscopic retrograde cholangiopancreatography

**DOI:** 10.1371/journal.pone.0207539

**Published:** 2018-11-19

**Authors:** Shiro Hayash, Tsutomu Nishida, Tokuhiro Matsubara, Naoto Osugi, Aya Sugimoto, Kei Takahashi, Kaori Mukai, Dai Nakamatsu, Masashi Yamamoto, Koji Fukui, Masami Inada

**Affiliations:** 1 Department of Gastroenterology and Hepatology, Toyonaka Municipal Hospital, Toyonaka, Japan; 2 Department of Gastroenterology and Internal Medicine, Hayashi Clinic, Osaka, Suita, Japan; North Shore Long Island Jewish Health System, UNITED STATES

## Abstract

**Introduction:**

Various endoscopic procedures under fluoroscopic guidance are being rapidly adopted, and radiation exposure is considered to be increasing. However, there is little concern about this issue in gastroenterology practice. This study aims to evaluate the actual radiation exposure dose (RD) during endoscopic retrograde cholangiopancreatography (ERCP) and the factors affecting the RD.

**Methods:**

In this retrospective, single-center cohort study of 1157 consecutive patients who underwent ERCP between October 2012 and February 2017, we analyzed the influences of patient characteristics, procedure time (min), total fluoroscopy time (min), type of processing engine, experience of the endoscopist, and type of disease on the total RD (mGy).

**Results:**

The median procedure times were 28 min for common bile duct stones (CBDS), 25 min for distal malignant biliary obstruction (MBO), and 30 min for proximal MBO. Similarly, the median fluoroscopy times were 10.3, 8.8, and 13.4 min, and the median RDs were 167, 123, and 242 mGy, respectively. Proximal MBO required significantly longer procedure time and fluoroscopy time and resulted in greater RD than distal MBO (P = 0.0006, <0.0001, <0.0001) and CBDS (P = 0.015, <0.0001, <0.0001). Multiple linear regression showed that distal MBO and a novel processing engine negatively correlate with RD (P = 0.04, <0.0001) and that proximal MBO positively correlates with RD (P = 0.0001).

**Discussion:**

Procedure time and fluoroscopy time were significantly longer for proximal MBO than for CBDS and distal MBO. The type of disease and processing engine significantly influenced the RD during ERCP.

## Introduction

Demands for minimally invasive procedures for medical purposes are rapidly increasing. In the field of digestive endoscopy, various endoscopic procedures under X-ray fluoroscopic guidance are being adopted, such as endoscopic retrograde cholangiopancreatography (ERCP), interventional endoscopic ultrasonography (EUS), enteral endoscopy, and stenting [1] [2]. At the same time, increased radiation exposure in medical imaging has led to major concerns in society because of its potential harmful health effects, including cancer risk [3] [4] [5]. The International Commission on Radiological Protection (ICRP), the International Atomic Energy Agency (IAEA), and the United Nations Scientific Committee on the Effects of Atomic Radiation (UNSCEAR) recommend establishing guidance levels or diagnostic reference levels (DRLs), which are used in medical imaging with ionizing radiation, including roentgenography, computed tomography, and scintigraphy, to indicate whether the patient dose and administered activity (amount of radioactive material) are unusually high or low for a given procedure [6] [7]. DRL settings in each region are now spreading worldwide [8] [9] [10] [11]. However, DRLs for specific endoscopy procedures remain limited and are briefly described for ERCP in only the European Commission [8]. From the gastroenterology societies’ point of view, the European Society of Gastrointestinal Endoscopy (ESGE) 2012 guidelines recommended reporting patient radiation exposure dose (RD) in a national database, though the guidelines also stated that limited information is available regarding DRLs for ERCP [12]. The American Society for Gastrointestinal Endoscopy (ASGE) recommended the frequency with which fluoroscopy time and RD are measured and documented as quality indicators for ERCP [13]. However, there are few reports about RD in endoscopic procedures, though many studies have been reported from radiological and cardiovascular societies [14] [15]. Gastroenterological societies are about to address this issue [15] [16]. Therefore, we must observe the radiation exposure and reduce it to the lowest level to allow the procedure to be completed in a safe and timely manner. To accomplish this goal, endoscopists must collect these data in a national database and revise the DRLs for each fluoroscopic endoscopy procedure.

The aim of this study is to measure the actual RD and to evaluate the factors that may affect RD during ERCP.

## Material and methods

**Fig 1:**
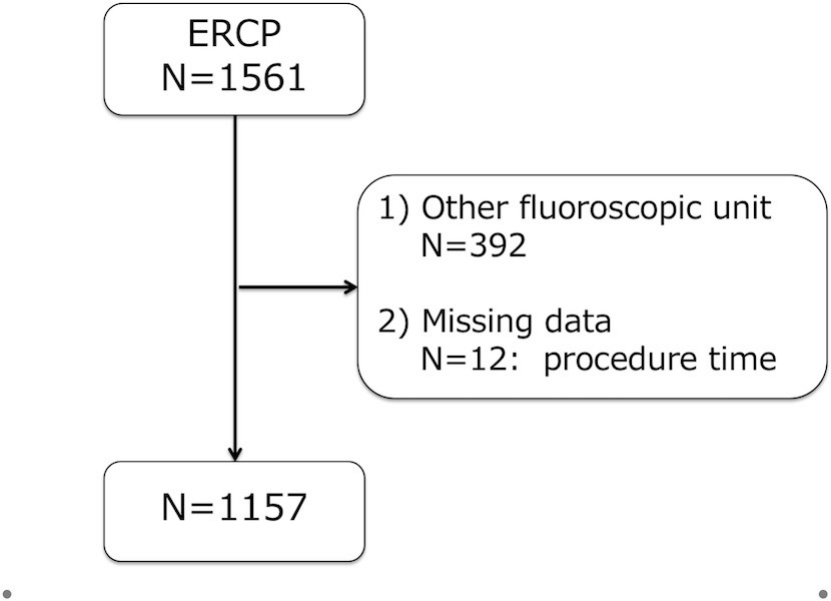
Study flow chart.

This is a retrospective, single-center cohort study of consecutive patients who underwent ERCP between October 2012 and February 2017 at the Toyonaka Municipal Hospital, which is certified as a teaching hospital by the Japan Gastroenterological Endoscopy Society (JGES; No. 1239). As a quality indicator for ERCP at our hospital, we have reported that the rate of post-ERCP pancreatitis was 3.9% (95% confidence interval: 3.02–5.07%) [17]. All the procedures were performed using wire-loaded cannulation method, which includes both contrast method and wire-guided method. During the study period, a total of 1561 ERCP procedures were performed using our fluoroscope unit. Among them, 392 procedures that were not performed with the EXAVISTA 17 fluoroscope unit (Hitachi, Japan) were excluded to evaluate the RD under the same circumstances, and 12 procedures were excluded due to missing procedure time data. Finally, 1157 total procedures were analyzed (Fig 1).

We examined the procedure time (min), total fluoroscopy time (min), radiation dose (mGy), radiation dose rate (RDR, mGy/min), and dose area product: DAP (mGycm^2^) during ERCP. We evaluated the following factors: age, gender, native papilla, disease requiring ERCP, experience of the endoscopist, and type of processing engine, all of which potentially affect the RD. The present study was conducted in accordance with the Declaration of Helsinki, and approval was obtained from the Institutional Review Board (2014-02-04).

### Definitions of the factors

Procedure time was defined from when we viewed the papilla to when we removed the endoscope from the patient. The fluoroscopy time and RD were automatically recorded in each procedure by the fluoroscope unit. The diseases were classified into the following 4 types: 1) common bile duct stones (CBDS); 2) distal malignant biliary obstruction (MBO), including obstruction caused by pancreatic cancer, ampullary cancer, distal CBD cancer, intraductal papillary mucinous cancer (IPMC), and duodenal cancer; 3) proximal MBO, including obstruction caused by upper CBD cancer, intrahepatic BD cancer, gall bladder cancer, hepatocellular carcinoma, and metastasis; and 4) other diseases. We categorized the experience of the endoscopist into 2 groups: high-volume endoscopists (HVEs), consisting of endoscopists with over 10 years of experience, approximately 200 ERCP procedures in the preceding year, and licensed as a board-certified fellow of the JGES; and low-volume endoscopists (LVEs), consisting of all other endoscopists, even considering the following strategy. We conducted this study based on routine clinical practice in an educational hospital certified by the JGES. Therefore, LVEs were often replaced by HVEs to address difficulties.

All procedures were performed under the same EXAVISTA 17 fluoroscope unit (Hitachi, Japan), including 2 image processing engines. We then classified the procedures into 2 groups depending on the image processing engines: ‘FAICE-V’ (a previous version of the processing engine, Hitachi, Japan) and ‘FAICE-V New Stage 1’ (NS1, a novel processing engine, Hitachi, Japan), which has been utilized in our hospital since July 2016 in the last 8 of the 52 months of the study period (52 months). NS-1 basically used three image processing technologies (frame rate conversion, motion tracking noise reduction, and multidynamic range compression) and was improved to complement the image deterioration of pulsed images and to keep the pulse rate low to maintain a lower RD than the RD with the previous version of the processing engine.

### Statistical analysis

All of the continuous variables are expressed as means±standard deviations (SDs) except for the nonparametric variables, which are expressed as medians and ranges. The categorical variables are expressed as numbers in each category or frequencies. The continuous variables were compared using Student’s t-tests or Dunn’s multiple comparison tests. The categorical variables were compared using chi-square tests or Fisher’s exact tests when appropriate. Simple linear regression analysis was performed to identify the relationships of procedure time with fluoroscopy time and RD. Multiple linear regression analysis was performed to identify the factors related to RD. Age, female sex, distal MBO, proximal MBO, CBDS, native papilla, HVE, and NS1 were included as covariates with RD. A P value of 0.05 was considered to indicate statistical significance. All statistical analyses were performed with the use of JMP software (ver. 13.1.0, SAS Institute, Inc., Cary, NC, USA).

## Results

### Patient characteristics

The patient characteristics are summarized in Table 1. A total of 1157 procedures were analyzed in the present study. The mean age was 73.6±10.8 years, and 490 patients were female (42%). Five hundred fifty patients (48%) had a native papilla, and post-ERCP pancreatitis occurred in 39 patients (3.4%). ERCP procedures were performed for the purpose of treating CBDS (N = 579, 50%), distal MBO (N = 224, 19%), proximal MBO (N = 240, 21%), or other diseases (N = 114, 10%).

**Table 1:** Characteristics of all patients and procedures.

Variable	Total (N = 1157)
Age (years), mean±SD	73.6±10.8
Female sex, %	490, 42
Native papilla, %	550, 48
PEP*, %	39, 3.4
**Disease site**	
CBDS†, %	579, 50
Distal MBO‡, %	224, 19
Proximal MBO, %	240, 21
NS1§, %	171, 15
Performed by an HVE||, %	610, 53
Procedure time (min), median [IQR]	28, [17–43]
Fluoroscopy time (min), median [IQR]	10, [6–17]
Radiation dose (mGy), median [IQR]	167, [90–307]
Dose Area Product (Gycm^2^), median [IQR]	18.1, [10.2–31.3]
Radiation dose rate (mGy/min), median [IQR]	17, [12–21]

* PEP: Post-endoscopic retrograde cholangiopancreatography pancreatitis

† CBDS: Common bile duct stones

‡ MBO: Malignant biliary obstruction

§ NS1: New Stage 1 (a novel processing engine of the ‘EXAVISTA’ fluoroscope unit)

|| HVE: High-volume endoscopist

### Procedure time and RD

**Fig 2:**
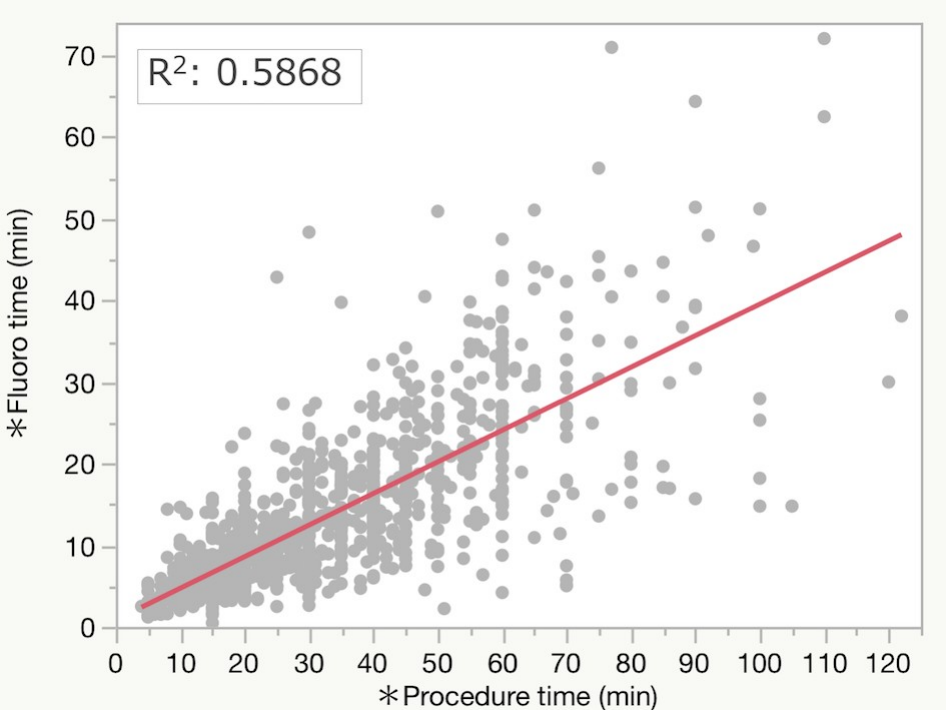
Simple linear regression test comparing fluoroscopy time and procedure time.

**Fig 3:**
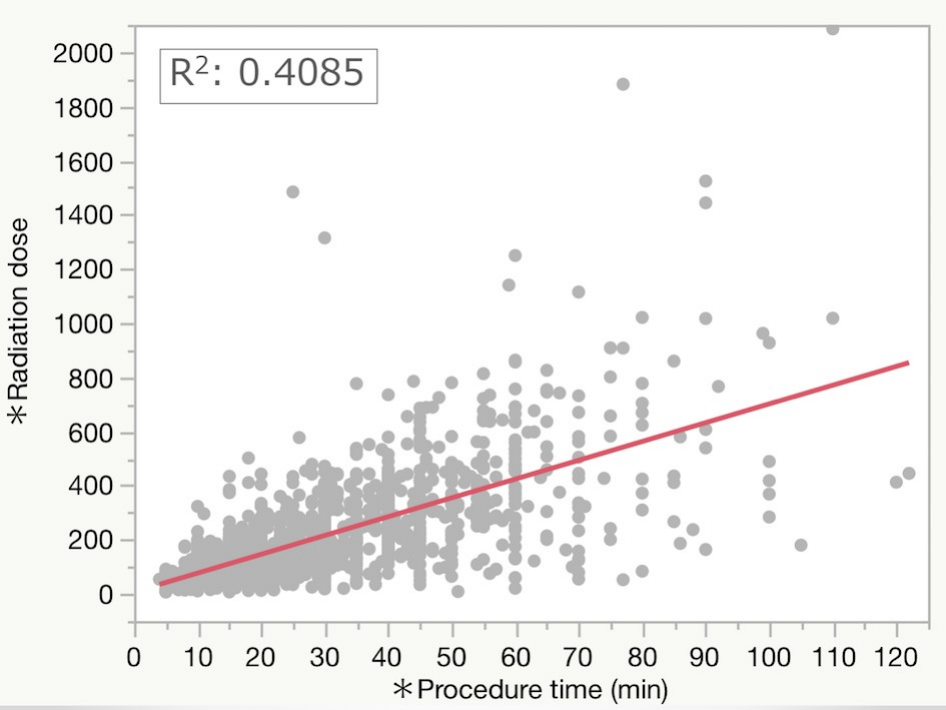
Simple linear regression test comparing radiation dose and procedure time.

The median procedure time (interquartile range: IQR) and fluoroscopy time were 28 (17–43) min and 10 (6–17) min, respectively. The median RD was 167 (90–306) mGy. The RDR was 17 (12–21) mGy/min. The median DAP was 18,138 (10,189–31,326) mGycm^2^. In the simple linear regression, procedure time was significantly positively correlated with fluoroscopy time (R^2^: 0.5868, P<0.0001, Fig 2) and with RD (R^2^: 0.4085, P<0.0001, Fig 3).

### Disease site

**Fig 4:**
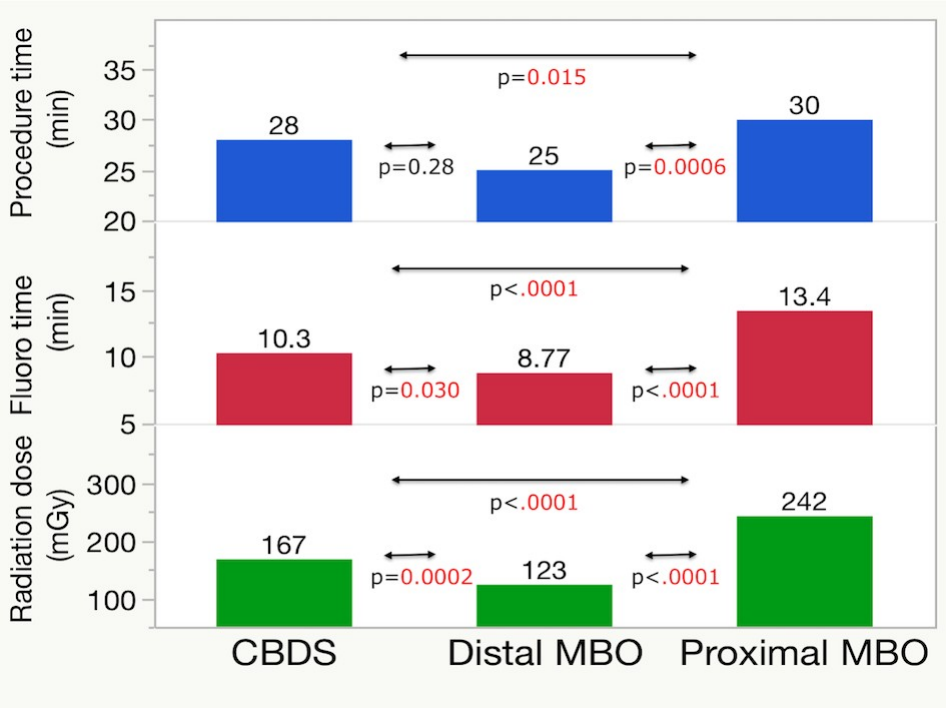
Time and dose: Multiple comparisons among disease sites.

The median procedure times among the three diseases were 28 (12–42) min for CBDS, 25 (15–40) min for distal MBO, and 30 (20–46) min for proximal MBO. Similarly, the median fluoroscopy times were 10 (6–17), 9 (4–13), and 13 (5–21) min, respectively. The median RDs were 167 (92–305), 123 (72–221), and 242 (124–410) mGy, respectively (Table 2). Dunn’s multiple comparison test among these three disease types revealed that proximal MBO showed significantly longer procedure and fluoroscopy times and a greater RD than both distal MBO (P = 0.0006, <0.0001, <0.0001) and CBDS (P = 0.015, <0.0001, <0.0001). CBDS showed a significantly longer fluoroscopy time and a greater RD than distal MBO (P = 0.030, 0.0002, Fig 4).

**Table 2:** Times and doses among disease sites.

	CBDS† N = 579	Distal MBO‡ N = 224	Proximal MBO N = 240
Procedure time (min), median [IQR*]	28 [12–42]	25 [15–40]	30 [20–46]
Fluoroscopy time (min), median [IQR]	10 [6–17]	9 [4–13]	13 [5–21]
Radiation dose (min), median [IQR]	167 [92–305]	123 [72–221]	242 [124–410]

* IQR: Interquartile range

† CBDS: Common bile duct stones

‡ MBO: Malignant biliary obstruction

### Factors affecting RD

Multiple linear regression analysis revealed that distal MBO and NS1 had a significantly negative relationship with RD (P = 0.04, <0.0001, respectively) and that proximal MBO had a significantly positive relationship with RD (P = 0.0001). Age, gender, CBDS, native papilla, and HVE did not have significant relationships with RD (Table 3).

**Table 3:** Factors affecting RD: Multiple linear regression analysis.

Variable	T value	P value
Age	0.3	0.7
Female	-1.8	0.08
Distal MBO*	-2.1	0.04
Proximal MBO	3.9	0.0001
CBDS†	0.5	0.6
Native papilla	-0.3	0.80
HVE‡	-1.2	0.3
NS1§	-7.2	<0.0001

* MBO: Malignant biliary obstruction

† CBDS: Common bile duct stones

‡ HVE: High-volume endoscopist

§ NS1: New Stage 1 (a novel processing engine of the ‘EXAVISTA’ fluoroscope unit)

## Discussion

The trend of increasing radiation exposure in medical imaging has led to major concerns in society because of its potential cancer risk [3] [4] [5]. For a long time, radiation exposure had been thought to have a threshold for inducing carcinogenic effects. However, recent studies have revealed that even low-dose exposure to radiation has carcinogenic effects as a stochastic effect [18] [19]. Moreover, the radiation exposure from medical procedures is increasing, and it is estimated that approximately 48% of the effective dose was delivered to the U.S. population in 2006, increasing from 15% in the early 1980s according to the National Council on Radiation Protection and Measurements (NCRP) report number 160 [20]. Computed tomography accounted for approximately half of this radiation. Next, fluoroscopic procedures and nuclear medicine accounted for approximately one-quarter. Therefore, all the radiation use in medical imaging, including fluoroscopic procedures, must be measured and managed [21] because of the carcinogenic effects of radiation exposure. Many registries and suggestions from both radiologic and cardiovascular societies were announced [15] [14] [22] [23]. However, gastroenterologists seem less interested in this issue than other medical societies, such as radiologists and cardiologists [15] [16]. Among the gastroenterological societies, the European Society of Gastrointestinal Endoscopy (ESGE) published guidelines in 2012 [12], and then, the ASGE and the American College of Gastroenterology (ACG) presented the Quality Indicators for ERCP in 2015 [13]. The guidelines recommend RD and fluoroscopy time as indicators, but there were no concrete numerical references, such as DRLs, and the level of evidence was 2+ or 2C (weak recommendation).

Until now, the DRL settings have not covered enough endoscopic procedures under fluoroscopic guidance. The ESGE guidelines say that the mean radiation dose (entrance surface dose, ESD) during ERCP was between 55 and 347 mGy from only 608 procedures of 7 studies [12], but the ESD ranged widely, and information regarding the DRLs for ERCP is quite limited. Similarly, many gastroenterology societies did not describe the DRL settings in their guidelines. In the present study, we examined the median RD during ERCP from approximately double the number of procedures in a single center compared with the total number of procedures previously reported in 7 studies from the ESGE guidelines. In addition, we clearly revealed that the radiation exposure varied widely depending on the disease site and the type of fluoroscopic system, including the processing engine.

To manage RD, it is important to know the current RD. The ASGE and ACG stated that the fluoroscopy time is practical to use as a surrogate marker of RD and should be recorded for all ERCP cases [13]. In the present study, the RDRs were 17.7 mGy/min with the previous system and 9.7 mGy/min with NS1. We clearly showed that different processing engines deliver different RDs (S1 Table). Those data suggest that the RDR for ERCP was not very high compared with the DRL for interventional radiology (IVR) in Japan of 20 mGy/min, which was calculated from the 87th percentile of 320 Japanese institutions [11]. In particular, a new processing engine can significantly reduce the total RD (P<0.0001). It is well known that frame rate directly correlates with RD and that half pulsed imaging reduces the RD [24] [25]. NS1 utilizes half pulsed imaging, and this effect of RD reduction seems to be natural, as described in other reports [26] [27]. However, the important point is that NS1 contains some novel technologies that maintain image quality even in a half-frame rate [28] (S1 video). Recently, Hoffman et al. reported the RD reduction effect provided by noise reduction technology in cardiac device implantation [29]. Generally, low frame rates concurrently decrease image quality with regard to smoothness, sharpness, and persistence of vision. The lack of an objective scale for the quality of fluoroscopy imaging is problematic, as no such scale has been developed yet. A scale of image quality could define the lower limit of the frame rate for diagnosis and the lower limit of RD. We think it is necessary to establish an objective scale of image quality for discussion. We hope that advanced image-enhancing technology may reduce the RD and generate better images in the near future.

In clinical practice, we often experience various difficulties and procedure times during ERCP procedures. Choi MH et al. reported that the location of disease influenced the RD in 217 ERCP procedures [30]. In the present study, conducted in 1157 cases, our results also showed that the type of disease affects the RD; specifically, proximal MBO required greater RD than distal MBO and CBDS. This recognition of practical doses is necessary in managing the RD of each ERCP procedure.

There are some reports about the positive relation between the physician’s experience and RD reduction [31] [32] [33]. HVEs can perform shorter procedure times than LVEs; a shorter procedure time results in a shorter fluoroscopy time; and finally, a shorter fluoroscopy time results in a lower RD. However, this study did not show a significant difference between HVEs and LVEs. The reasons could be that an LVE must perform ERCP with an HVE in our institution and that LVEs were often replaced by HVEs to address their difficulties. Second, HVEs performed more difficult ERCP procedures, such as proximal MBO or native papilla, than LVEs did. As a result, there were no significant differences in post-endoscopic retrograde cholangiopancreatography pancreatitis (PEP), fluoroscopy time, or RD between HVEs and LVEs (Table 4). In other words, this result means that adequate management of personnel might control for the negative effect of LVEs.

**Table 4:** Comparisons of characteristics between HVEs and LVEs.

Variables	HVEs (N = 604)	LVEs (N = 534)	P value
Age (years), mean±SD	73.2±10	74.2±11	0.03
Female, %	252, 41	235, 44	0.4
Native papilla, %	312, 51	232, 43	0.007
PEP*, %	22, 3.6	15, 2.8	0.4
Disease site			
CBDS†, %	261, 43	315, 59	<0.0001
Distal MBO‡, %	130, 21	93, 17	0.09
Proximal MBO, %	140, 23	97, 18	0.04
NS1§, %	88, 14	75, 14	0.8
Procedure time (min)	30	25	0.0002
Fluoroscopy time (min)	11	10	0.09
Radiation dose (mGy)	167	169	0.9

* PEP: Post-endoscopic retrograde cholangiopancreatography pancreatitis

† CBDS: Common bile duct stones

‡ MBO: Malignant biliary obstruction

§ NS1: New Stage 1 (a novel processing engine of the ‘EXAVISTA’ fluoroscope unit)

There is no limit or benchmark for fluoroscopy time, which depends on the endoscopist. However, it may be useful for us to refer to the DRL for IVR as a guide. This study also revealed the correlations of procedure time with fluoroscopy time and RD. Therefore, similar to the fluoroscopy time, the procedure time can be a quality indicator of ERCP. In the future, we will set DRLs to manage and reduce the RD during various fluoroscopic procedures. It will then be necessary to know the RD and fluoroscopy time in each institution. Moreover, endoscopists and gastroenterological societies must correct those data, set an optimal DRL, and educate the endoscopists who do not normally receive formal training in operating fluoroscopy machines or in minimizing their own radiation exposure [34] [35].

## Supporting information

S1 Table Characteristics between the NS1 and Previous groups. This is the S1 Table Title.
(DOCX)

S1 Video This is the S1 Video Title. (MP4)
