# Correction: Radiation exposure dose and influencing factors during endoscopic retrograde cholangiopancreatography

**DOI:** 10.1371/journal.pone.0209877

**Published:** 2018-12-20

**Authors:** Shiro Hayashi, Tsutomu Nishida, Tokuhiro Matsubara, Naoto Osugi, Aya Sugimoto, Kei Takahashi, Kaori Mukai, Dai Nakamatsu, Masashi Yamamoto, Koji Fukui, Masami Inada

The first author's name is spelled incorrectly. The correct name is: Shiro Hayashi. The correct citation is: Hayashi S, Nishida T, Matsubara T, Osugi N, Sugimoto A, Takahashi K, et al. (2018) Radiation exposure dose and influencing factors during endoscopic retrograde cholangiopancreatography. PLoS ONE 13(11): e0207539. https://doi.org/10.1371/journal.pone.0207539.
