# Supplementary material for: Radiation exposure dose and influencing factors during endoscopic retrograde cholangiopancreatography
Source: PLoS One. 2018 Nov 19;13(11):e0207539. doi: 10.1371/journal.pone.0207539 (PMC6242372; doi:10.1371/journal.pone.0207539)
Supplement: S1 Table — This is the S1 Table Title. (DOCX) [file pone.0207539.s001.docx]

**S1 Table. Characteristics between the NS1 and Previous groups**

| **Variables** | **NS1 (N=171)** | **Previous (N=986)** | **P value** |
| --- | --- | --- | --- |
| Age (years), mean±SD | 73.1±11 | 73.7±11 | 0.6 |
| Female, % | 77, 45 | 413, 42 | 0.44 |
| Native papilla, % | 95, 56 | 455, 46 | 0.02 |
| PEP*, % | 7, 4.1 | 32, 3.3 | 0.6 |
| Disease site |  |  |  |
| CBDS†, % | 103, 43 | 476, 48 | 0.005 |
| Distal MBO‡, % | 21, 12 | 203, 21 | 0.01 |
| Proximal MBO, % | 25, 15 | 215, 22 | 0.03 |
| Procedure time (min) | 25 | 30 | 0.01 |
| Fluoroscopy time (min) | 9 | 11 | 0.002 |
| Radiation dose (mGy) | 93 | 186 | <.0001 |
| Radiation dose rate (mGy/min) | 9.7 | 17.7 | <.0001 |

* PEP: Post-endoscopic retrograde cholangiopancreatography pancreatitis

† CBDS: Common bile duct stones

‡ MBO: Malignant biliary obstruction

§ NS1: New Stage 1 (a novel processing engine of the ‘EXAVISTA’ fluoroscope unit)
